# Subsequently occurring bilateral iliopsoas hematoma: a case report

**DOI:** 10.1186/s13019-015-0386-7

**Published:** 2015-12-11

**Authors:** Kyo Seon Lee, In Seok Jeong, Sang Gi Oh, Byung Hee Ahn

**Affiliations:** Department of Thoracic and Cardiovascular Surgery, Chonnam National University Hospital, Chonnam National University School of Medicine, 42, Jebong-ro, Gwangju, Dong-gu 501-757 South Korea

**Keywords:** Iliopsoas hematoma, Anticoagulant, Endovascular procedures

## Abstract

**Background:**

Spontaneous bilateral iliopsoas hematomas is a rare complication after anticoagulant therapy. Furthermore, the onset of bilateral iliopsoas hematoma is unknown because the causes are unclear.

**Case Presentation:**

A 65-year-old man on anticoagulant therapy after mechanical aortic valve replacement was admitted after presenting with severe pain in the left flank and abdomen. Abdominal CT revealed a large left-sided iliopsoas hematoma with extravasation. Fresh frozen plasma was transfused due to prolonged prothrombin time. Transarterial embolization was successfully performed. During the hospital stay, follow-up abdominal CT was performed and a small right-sided iliopsoas hematoma was detected. This was closely observed and an intervention was not performed, as the patient was asymptomatic. The final CT prior to discharge revealed a reduction in size of each hematoma.

**Conclusions:**

Spontaneous bilateral iliopsoas hematoma can be developed subsequently. Patients with unilateral iliopsoas hematoma should be closely monitored for development of bilateral iliopsoas hematoma.

## Background

Anticoagulant therapy after valvular surgery is associated with various bleeding events. These complications can cause hospitalization, death, permanent injury, or necessitate transfusion of blood products. This occurs at an incidence of up to 30 % [1, 2].

Although there have been some reports of unilateral iliopsoas hematomas, only few cases of bilateral hematomas have been reported; therefore, its incidence remains unclear. To the best of our knowledge, this is the first reported case of bilateral iliopsoas hematomas that involved a spontaneous hematoma with the subsequent development of a second hematoma.

## Case presentation

A 65-year-old man presented with escalating dull pain to the left flank and lower back for 2 days. In addition, he experienced abdominal pain just before the admission. Surgical history included a mechanical aortic valve replacement 6 months ago. The patient was taking a vitamin K antagonist. He reported no history of trauma for several months prior to admission. There was no familial tendency toward bleeding. On admission, his heart rate was 110 beats per minute with sinus rhythm and blood pressure was 110/70 mmHg.

Ecchymotic lesion was noted on his left flank and he experienced tenderness of the left flank and left lower quadrant of the abdomen. There was no rebound tenderness though the patient expressed that the pain worsened when bending over. The results of laboratory test revealed decreased hematocrit level at 23.8 % and prolonged international normalized ratio of prothrombin time at 5.25. The platelet count was within normal range.

**Fig. 1:**
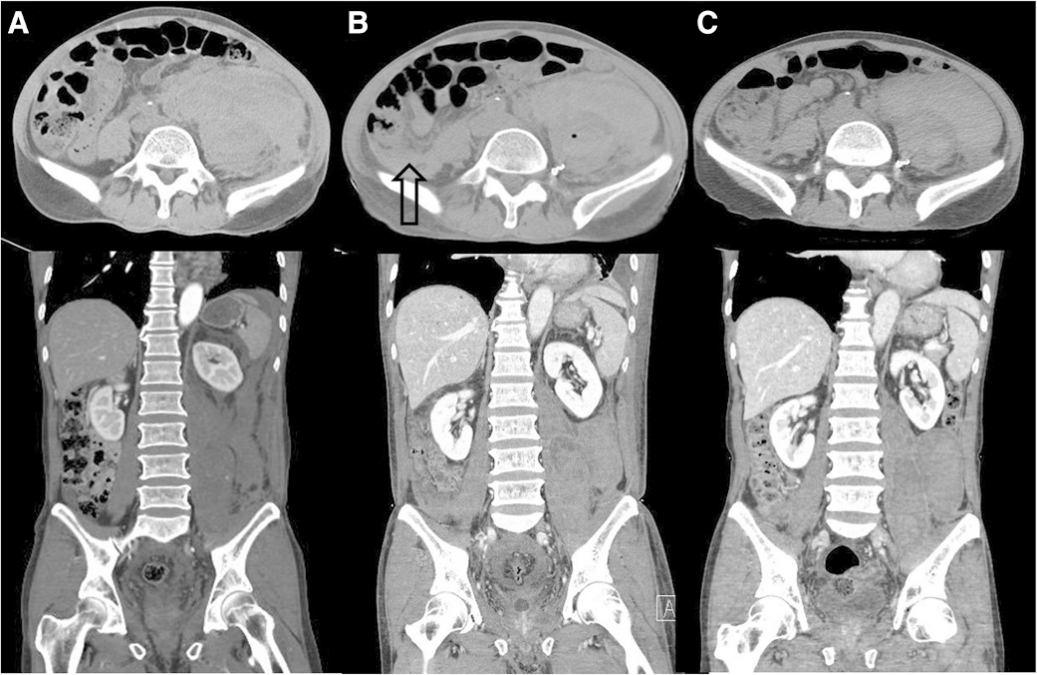
Serial abdominal CT. **a**: Initial abdominal CT; **b**: Five days after TAE, the arrow indicates the newly developed right iliopsoas hematoma; **c**: Final follow-up CT, CT : computed tomography, TAE : transarterial embolization

**Fig. 2:**
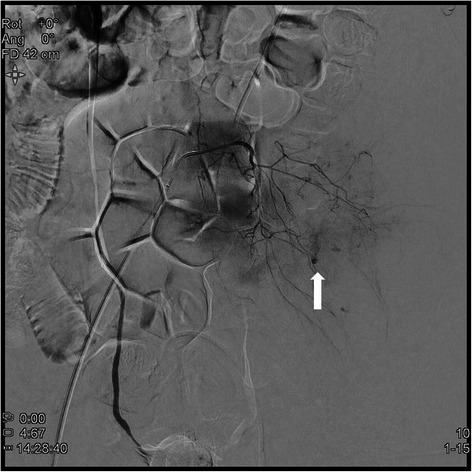
The arrow indicates active bleeding in branches of left 5th lumbar artery

Abdominal computed tomography (CT) was performed to evaluate the abdominal and left flank pain and revealed a large hematoma of the left iliopsoas muscle and extravasation of contrast material. Hemoperitoneum was also revealed though the amount was minimal (Fig. 1a). The patient continued to have abdominal pain and follow-up hematocrit level dropped to 21.5 %. Fresh frozen plasma was infused and an emergent lumbar arterial angiogram was performed for hemostasis. There was active bleeding on the left 5th lumbar arterial branch. Transarterial embolization (TAE) was successfully performed (Fig. 2).

On day 5 of hospitalization, follow-up abdominal CT was performed. It revealed decrease in the size of the hematoma in the left iliopsoas muscle. However, a new small hematoma was detected in the right iliopsoas muscle (Fig. 1b). Close observation was the plan of care for this finding as it was not large enough to cause symptoms. Furthermore, there was no extravasation of contrast media in the right iliopsoas hematoma. Follow-up abdominal CT was performed on day 12 of hospitalization and showed decrease in the size of two retroperitoneal hematomas that had become more liquefied. The hemoperitoneum was no longer evident (Fig. 1c). Fortunately, the patient did not experience worsening symptoms associated with the hematoma and the hematocrit concentration was constant without the need for additional transfusion during hospitalization.

We adjusted prothrombin time international normalized ratio from 2.0 to 2.5 and he was discharged without complications.

Iliopsoas hematoma has been reported to occur mainly in relation to trauma; however, spontaneous iliopsoas hematoma has been reported in patients with coagulation problems, such as hemophilia, liver cirrhosis, and anticoagulants-related incidents [3–6].

The mechanism of spontaneous iliopsoas hematoma is unclear, but tearing of muscle fibers, small vessel atherosclerosis, heparin-induced immune microangiopathy, and unrecognized minor trauma are generally accepted etiologies [7, 8]. The exact mechanism of the bilateral iliopsoas hematoma is more difficult to determine, as in this case, it could be assumed that the unilateral hematoma occurs first and the opposite later. Although it is unlikely that muscle tearing would occur at the same time, if the patient were to use the contralateral leg to relieve ipsilateral leg pain, muscle tearing is a possible explanation for the etiology of spontaneous bilateral iliopsoas hematoma.

Iliopsoas hematoma has a variety of clinical manifestations. The most common symptom is sudden onset of lower back and flank pain. Patients also describe using leg flexion to try to relieve pain on the involved side (psoas sign). They may also present with ecchymotic lesions on the affected flank, the so-called Grey Turner’s sign, which is a result of massive retroperitoneal hematomas, as with our patient. A large amount bleeding may cause hypovolemic shock and abdominal compartment syndrome [7]. Iliopsoas hematomas have also been associated with compressive femoral neuropathy, because the nerve can be compressed anywhere along its course. Femoral neuropathy and motor weakness of the lower extremity, develops in almost all patients [3, 4, 6].

There is no clear strategy for treatment of iliopsoas hematoma yet, and a variable treatment plan may be used according to the patient’s condition. Close monitoring is required when administering anticoagulants to prevent the possibility of complications occurring as a result of anticoagulant overuse [6]. However, despite conservative management, if fatal complications occur—such as femoral neuropathy, hypovolemic symptom or abdominal compartment syndrome—aggressive surgical intervention should be considered [4, 7].

Although TAE is safer, faster, and less invasive than surgical intervention for controlling active bleeding, the hematoma cannot be evacuated in this manner. Therefore, surgical intervention should be considered preferentially when femoral neuropathy or abdominal compartment syndrome develops due to mass effect of the large hematoma [3, 5, 7]. Our patient underwent TAE for two reasons. First, he needed immediate hemostasis because he was exhibiting hypovolemic signs that led to the development of femoral neuropathy or abdominal compartment syndrome. Second, the bleeding had to be controlled in order to reinitiate the anticoagulant therapy for preventing prosthetic valvular thrombi. The newly developed right iliopsoas hematoma that occurred during the hospital stay was managed with conservative treatment because it was small and the patient was asymptomatic.

## Conclusions

This case may be the first report about spontaneous bilateral iliopsoas hematoma that developed subsequently. Patients treated with anticoagulant therapy should be closely monitored for development of abdominal or flank pain to rule out retroperitoneal hematoma. In addition, patients with unilateral iliopsoas hematoma could also develop bilateral hematoma.

## Consent

Written informed consent was obtained from the patient for publication of this Case report and any accompanying images. A copy of the written consent is available for review by the Editor-in-Chief of this journal.

## Abbreviations

CT: computed tomography
TAE: transarterial embolization
